# The Absence of the Transcription Factor Yrr1p, Identified from Comparative Genome Profiling, Increased Vanillin Tolerance Due to Enhancements of ABC Transporters Expressing, rRNA Processing and Ribosome Biogenesis in *Saccharomyces cerevisiae*

**DOI:** 10.3389/fmicb.2017.00367

**Published:** 2017-03-16

**Authors:** Xinning Wang, Zhenzhen Liang, Jin Hou, Yu Shen, Xiaoming Bao

**Affiliations:** ^1^State Key Laboratory of Microbial Technology, School of Life Science, Shandong UniversityJinan, China; ^2^Shandong Provincial Key Laboratory of Microbial Engineering, Department of Bioengineering, Qilu University of TechnologyJinan, China

**Keywords:** budding yeast, vanillin tolerance, *YRR1*, *ADH7*, ABC transporters, rRNA processing, ribosome synthesis

## Abstract

Enhancing the tolerance of *Saccharomyces cerevisiae* to inhibitors derived from lignocellulose is conducive to producing biofuel and chemicals using abundant lignocellulosic materials. Vanillin is a major type of phenolic inhibitor in lignocellulose hydrolysates for *S. cerevisiae*. In the present work, the factors beneficial to vanillin resistance in yeast were identified from the vanillin-resistant strain EMV-8, which was derived from strain NAN-27 by adaptive evolution. We found 450 SNPs and 44 genes with InDels in the vanillin-tolerant strain EMV-8 by comparing the genome sequences of EMV-8 and NAN-27. To investigate the effects of InDels, InDels were deleted in BY4741, respectively. We demonstrated that the deletion of *YRR1* improved vanillin tolerance of strain. In the presence of 6 mM vanillin, deleting *YRR1* increase the maximum specific growth rate and the vanillin consumption rate by 142 and 51%, respectively. The subsequent transcriptome analysis revealed that deleting *YRR1* resulted in changed expression of over 200 genes in the presence of 5 mM vanillin. The most marked changes were the significant up-regulation of the dehydrogenase *ADH7*, several ATP-binding cassette (ABC) transporters, and dozens of genes involved in ribosome biogenesis and rRNA processing. Coincidently, the crude enzyme solution of BY4741(*yrr1*Δ) exhibited higher NADPH-dependent vanillin reduction activity than control. In addition, overexpressing the ABC transporter genes *PDR5, YOR1*, and *SNQ2*, as well as the RNA helicase gene *DBP2*, increased the vanillin tolerance of strain. Interestingly, unlike the marked changes we mentioned above, under vanillin-free conditions, there are only limited transcriptional differences between wildtype and *yrr1*Δ. This indicated that vanillin might act as an effector in Yrr1p-related regulatory processes. The new findings of the relationship between *YRR1* and vanillin tolerance, as well as the contribution of rRNA processing and ribosome biogenesis to enhancing *S. cerevisiae* vanillin tolerance, provide novel targets for genetic engineering manipulation to improve microbes’ tolerance to lignocellulose hydrolysate.

## Introduction

*Saccharomyces cerevisiae* is widely used in traditional ethanol production and shows promise as a cell factory for other chemical production because it is easily genetically manipulable, robust, and generally recognized as safe (Liu et al., 2009). In recent decades, much effort has been invested in producing ethanol and valuable chemicals with *S. cerevisiae* from lignocellulosic materials because these materials are the abundant renewable biomass resource on earth (Kim et al., 2015). The pretreatment process is critical for the effective use of lignocellulose because it is necessary to destroy the complicated structure of lignocellulose and to release monosaccharides. However, toxic chemicals, such as organic acids, furans, and phenolics, are unavoidably produced during pretreatment, and they inhibit the growth and metabolism of microorganisms in the subsequent fermentation process (Klinke et al., 2004; Liu, 2011). Among these toxic chemicals, the phenolics, which are produced from the segmental degradation of lignin, exhibit more toxicity than organic acids and furans to fermentation by *S. cerevisiae* (Klinke et al., 2004). Lignocellulose hydrolysate contains three kinds of phenolics, classified by the radicals they contain (para-hydroxyphenyl, guaiacyl, and syringyl). Vanillin is a typical guaiacyl phenolic (Klinke et al., 2004; Wang et al., 2016). It has been confirmed that in *S. cerevisiae*, vanillin triggers ROS accumulation, fractures the mitochondria and represses translation (Iwaki et al., 2013b; Nguyen et al., 2014a,b). To date, there are three strategies to improve the vanillin tolerance of *S. cerevisiae*. First, vanillin can be converted to the less-toxic vanillyl alcohol by certain reductases and dehydrogenases; therefore, identifying efficient reductases and dehydrogenases and overexpressing them has proven effective (Liu, 2011). Second, strains having higher ergosterol synthesis capacity show higher vanillin tolerance because higher levels of ergosterol enhance the fluidity and stability of the membrane (Endo et al., 2009). Finally, because vanillin causes cellular ROS accumulation (Nguyen et al., 2014a), high glutathione levels increase yeast robustness (Ask et al., 2013). Further studies are necessary to identify other effective strategies for improving the resistance of *S. cerevisiae* to vanillin.

In our previous study, a high vanillin-tolerant *S. cerevisiae* strain, EMV-8, was obtained via mutation induced by ethyl methanesulfonate (EMS) and then adaptive evolution in the hydrolysate. EMV-8 exhibited a high vanillin consumption rate and a high total antioxidant capacity (Shen et al., 2014). Furthermore, through transcriptome analysis of strain EMV-8 and subsequent gene overexpression tests, several dehydrogenases and reductases (*ZWF1, ALD6, YNL134C*, and *YJR096W*) were revealed to function in vanillin detoxification by converting vanillin to vanillyl alcohol (Wang et al., 2016). The functions of reductases and dehydrogenases in vanillin reduction were divided into two groups: ➀ have vanillin reduction activities (Adh6p, Adh7p, and proteins encoded by *YNL134C* and *YJR096W*); ➁ NADPH supply for vanillin reduction (Ald6p and Zwf1p). However, overexpressing these genes did not produce as much vanillin tolerance as observed in EMV-8. In this study, we sequenced the genome of EMV-8 and its parental strain, NAN-27. Several mutations were found. Among them, the absence of Yrr1p, a transcription factor, was demonstrated to improve tolerance to vanillin in strains with different genetic backgrounds. Then, the transcriptional differences between *yrr1*Δ and wildtype *S. cerevisiae* were compared to reveal the underlying mechanism.

## Materials and Methods

### Medium and Culture Conditions

Yeast extract peptone dextrose (YPD) medium (10 g L^-1^ yeast extract, 20 g L^-1^ tryptone, 20 g L^-1^ glucose) was used for activation, culture of host strains, and spot dilution growth (with 2% agar).

Synthetic complete medium (SD) or Sc-URA medium (1.7 g L^-1^ yeast nitrogen base, Sangon, China, 5 g L^-1^ ammonium sulfate, Sangon, China, CSM or CSM-URA, MP Biomedicals, Solon, OH, USA) was used for activation and batch fermentation of recombinant strains, supplying 20 g L^-1^ glucose. Vanillin was used as an inhibitor and was added in the medium as indicated. All of the cultures were grown at 30°C unless otherwise indicated.

### Strains and Plasmids

The *S. cerevisiae* industry strain NAN-27 (Zhang et al., 2010) and the vanillin-tolerant strain EMV-8, which was derived from NAN-27 (Shen et al., 2014), were the strains whose genomes were sequenced. Genetic manipulation including deletion and overexpression were mainly conducted in the lab strain BY4741 (*MATa, his3Δ1 leu2Δ met5Δ ura3Δ*, EUROSCARF, Germany). Deletion of *YRR1* was also conducted in CEN.PK2-1C (*MATa; ura3-52; trp1-289; leu2-3,112; his3Δ1; MAL2-8C; SUC2*, EUROSCARF, Germany). The genes for overexpression were amplified from the mutant strain EMV-8. *DBP2* was amplified from cDNA of BY4741. The gene deletion was performed using homologous recombination. The primers of this work are listed in Supplementary Table S1. The destruction cassette contained the *KanMX* expression cassette amplified from plasmid pUG6 (*Escherichia coli* plasmid with segment *LoxP–KanMX4–LoxP*) (Güldener et al., 1996) and the sequences that were homologous with the genes for knockout. The DNA fragments of the destruction cassettes were then transformed into the lab strain BY4741, and the mutants were screened in YPD medium with 800 mg L^-1^ G418. pJFE3 (Shen et al., 2012) is a 2 μ plasmid with the *TEF1* promoter, the *PGK1* terminator, and *URA3* as the selection marker. pJFE1 (developed in the laboratory) is a centromere plasmid with the *TEF1* promoter, the *PGK1* terminator, and the *URA3* expression cassette as the selection marker.

### Spot Dilution Growth Assay

A single colony was inoculated into 3 mL YPD medium and cultured for 12 h. The cultured cells were transferred into a fresh 10 mL of YPD medium with an initial OD_600_ of 0.2. After overnight culture, the cells were harvested and washed three times with ddH_2_O, followed by suspension in 1 mL of ddH_2_O for 9 h to consume endogenous substances. The suspended cells were normalized to an OD_600_ of 1.0. Then, 4 μl of 10-fold serial dilutions were spotted onto YPD or YPD with different concentrations of vanillin and incubated at 30°C for 1 days (for YPD) or 2–3 days (with vanillin in YPD).

### Fermentation

A single colony was cultured into 3 mL of SD or SC-URA for 24 h. Then, the cultures were transferred into 20 mL of fresh medium with OD_600_ at 0.2 for overnight culture. Then, the overnight-cultured cells were inoculated into 100-mL flasks with 40 mL of fermentation medium with an initial OD_600_ of 0.2. Then, the fermentation was performed at 30°C and 200 rpm.

### Calculation of Physiological Parameters

The biomass concentration was calculated with reference to the measured OD_600_-dry weight correlation. For BY4741, one unit of OD_600_ (detected by a BioPhotometer plus, Eppendorf, Germany) equals 0.18 g L^-1^ of biomass. The maximum growth rates were estimated as the linear regression coefficients of the ln (OD_600_) versus time during the exponential growth phase. Specific consumption rates of vanillin were calculated using the following equation:

$$r=\frac{A_{n}-A_{m}}{\frac{1}{2}\Sigma_{i=m+1}^{n}(B_{i}+B_{i-1})\times \left(t_{i}-t_{i-1}\right)}$$

where *r* is the specific consumption rate during the phase from sampling point *m* to sampling point *n*; *A*, *B*, and *t* are the metabolite concentration, biomass concentration, and time, respectively, at sampling points *n*, *i*, and *m*, as previously described (Peng et al., 2012).

### Analyses of Extracellular Fermentation Products

The concentrations of glucose and ethanol were measured using a Prominence LC-20A HPLC system (Shimadzu, Japan) furnished with an Aminex HPX-87H ion exchange column (Bio-Rad, USA) and refractive index detector RID-10A (Shimadzu, Japan), using 5 mmol L^-1^ H_2_SO_4_ as the mobile phase with a flow rate of 0.6 mL/min at 45°C (Eliasson et al., 2000). A BioSil-C18 column (Bio-Rad, USA) with a mobile phase of 40% aqueous methanol at a flow rate of 0.6 mL/min at room temperature was used to test the concentrations of vanillin and vanillyl alcohol by HPLC. The peaks were detected via ultraviolet detection (SPD-M20A) at 210 nm (Ji et al., 2011).

### Genome Sequencing

Genomic DNA was extracted from EMV-8 and NAN-27 using a yeast genome DNA extraction kit (Sangon Biotech, China) after yeast cells were harvested during the mid-log phase. Three paired-end genomic DNA libraries (200, 500, and 6000-bp) were constructed from NAN-27, and a 500-bp genomic DNA library was constructed from EMV-8. The library quality was assessed using the Qubit@ 2.0 Fluorometer (Thermo Scientific) and Agilent Bioanalyzer 2100 system. Finally, the library was sequenced on an Illumina HiSeq platform, and 125-bp paired-end reads were generated (an Illumina Hiseq2500 system).

### Transcriptome Analysis

The pre-cultured cells of BY4741 and BY4741(*yrr1*Δ) in SD medium were transferred into fresh SD or SD containing 5 mM vanillin with an initial OD_600_ of 0.2. The cells were harvested during the log phase (OD_600_≈1.6-2.0, vanillin content in the medium approximately 0.73∼0.78 g/L) and were quick frozen using liquid nitrogen. The UNIQ-10 Trizol RNA Purification Kit (Sangon Biotech, China) was used to extract total RNA. mRNA was isolated, fragmented, and then used as templates to synthesize cDNA. The short fragments were connected with adapters to obtain suitable fragments for PCR amplification. Ultimately, the libraries were sequenced by the Illumina HiSeq^TM^ 4000 (BGI Shenzhen, China). The differentially expressed genes were screened out according to the following criteria: Fold change ≥ 2 and FDR ≤ 0.001. All of the analyses were performed in biological duplicate. The transcriptome data were deposited in the NCBI Gene Expression Omnibus database (GEO accession number: GSE89854). Gene functions were annotated using the tool on *Saccharomyces genome database* (SGD)^1^.

### Enzyme Activity Assay

The strains were cultured in SD medium. The cells were collected when the OD_600_ reached 4.0. Then, the cells were disrupted using a Fast Prep cell homogenizer (Thermo Savant, Germany) with 33 mM Na_3_PO_4_ buffer (pH = 7.0) containing 0.5-mm glass beads and 1 mM PMSF, which was used to inhibit any protease activity. The supernatant was collected by centrifugation at 4°C for the enzyme activity assay (Larroy et al., 2002).

The vanillin dehydrogenase activity was measured using the assay described by Larroy (Larroy et al., 2002). The 0.6-mL reaction mixtures used to measure enzyme activities toward vanillin contained 33 mM sodium phosphate buffer (pH 7.0), 0.5 mM NADPH, and 1 mM vanillin (using cuvettes with 0.2-cm path length). The molar absorption coefficient (ε_365_) was 7.71 mM^-1^ cm^-1^ for vanillin plus NADPH. One unit (U) of enzyme activity is defined as the amount of enzyme that can reduce 1 μmol of NADPH plus vanillin in 1 min. The BCA protein assay reagent kit (Beyotime, China) was used to measure protein concentrations of crude enzyme solution. The specific enzyme activity (U mg^-1^ protein) refers to the enzyme activity per milligram of protein.

## Results

### Identification of the Mutations Affecting Vanillin Tolerance by Genome Sequencing and Growth Assay Screening

To identify the key genetic targets related to vanillin resistance, comparative genome analysis was performed between the vanillin-tolerant strain EMV-8 and its parent strain, NAN-27. The genome sequencing results identified a total of 450 non-synonymous SNPs in CDS and 44 CDS with InDels in strain EMV-8 compared to its parent strain, NAN-27. In the present work, we focused on genes with InDels. Using PCR amplification and sequence analysis, only seven genes with InDels were confirmed in the evolved strain (**Table 1**).

**Table 1:** Summary of InDels in the mutant strain EMV-8 compared with its parent strain, NAN-27.

Gene name	Mutations	Function
*MFG1*	Frameshift; damaged stop codon	Regulator of filamentous growth
*YNL195C*	Frameshift; deletion of 531 bp at the 3′ end	Protein of unknown function
*PAU17*	Frameshift; deletion of 91 bp within the gene	Protein of unknown function; member of the seripauperin multigene family encoded mainly in subtelomeric regions
*YVH1*	Deletion of 25 bp within the gene; truncation at 141 aa	Dual specificity protein phosphatase; regulates growth, sporulation, and glycogen accumulation in a cAMP-dependent protein kinase cascade dependent manner
*PPM2*	Frameshift; insertion of 6 bp within the gene; damaged stop codon	AdoMet-dependent tRNA methyltransferase
*YRR1*	Frameshift; truncation at 141 aa	Zn_2_-Cys_6_ zinc-finger transcription factor; activates genes involved in multidrug resistance
*RBG1*	Frameshift; deletion of 331 bp at the 5′ end; damaged start codon	Member of the DRG family of GTP-binding proteins; associates with translating ribosomes


**FIGURE 1:**
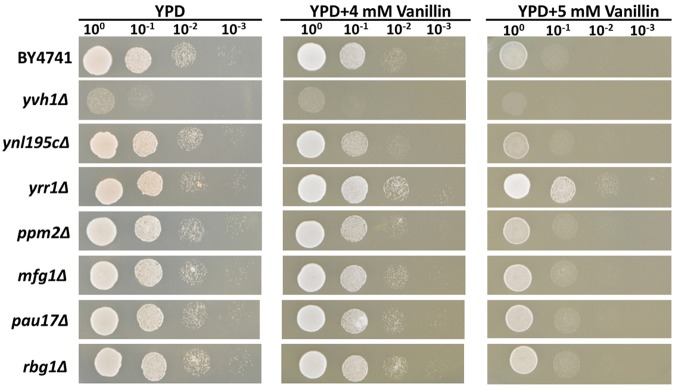
**Vanillin resistance profiles of BY4741 and its single-gene mutants.** The deletion mutants were derived from BY4741 and are shown with their genotypes. Four microliters of each 10-fold dilution with an initial cell OD_600_ = 1 was spotted on YPD (for 1 days) or YPD containing 4 or 5 mM vanillin (for 2–3 days).

To determine whether these InDel-containing genes influence vanillin tolerance in the mutant strain, these seven genes were separately knocked out in the lab strain BY4741. The growth profiles of the knock-out strains for these genes were tested using a spot dilution growth assay on media with or without vanillin (**Figure 1**).

### Nonsense Mutation of *YRR1* Increased the Vanillin Tolerance of *S. cerevisiae*, but Deleting its Paralog, *YRM1*, Did Not

**FIGURE 2:**
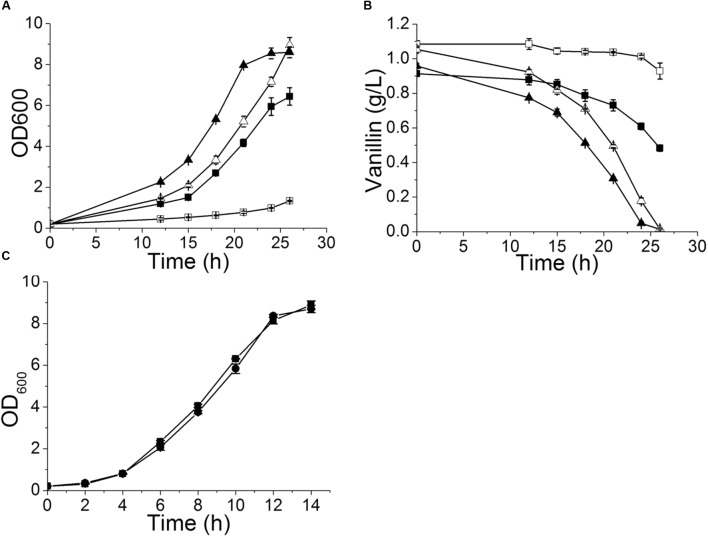
**Vanillin-resistance profiles of *yrr1*Δ and its parent strain in liquid medium.** The curves of growth **(A)** and vanillin consumption **(B)** when 5 or 6 mM vanillin was included in SD medium. Symbols: square, BY4741; triangle, BY4741(*yrr1*Δ); solid, SD with 5 mM vanillin; open, SD with 6 mM vanillin. **(C)** The growth curve in SD medium. Symbols: square, BY4741; circle, BY4741(*yrr1*Δ). The error bars indicate the standard deviation of independent triplicate experiments.

Deleting *YRR1*, as by the addition of a stop codon at 409 bp that shortened the final protein product from 811 to 141 aa in EMV-8, notably enhanced the strain growth on the plates containing vanillin (**Figure 1**). Meanwhile, deleting other genes did not improve the vanillin tolerance of the strain. The maximum specific growth rates of BY4741(*yrr1*Δ) were, respectively, 23 and 142% faster than those of BY4741 with 5 and 6 mM vanillin in SD medium (**Figure 2** and **Table 3**).

**FIGURE 3:**
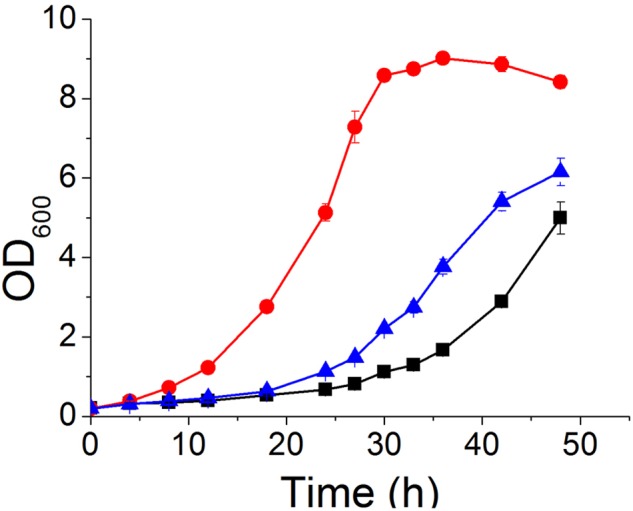
**Vanillin tolerance profiles of BY4741(pJFE1) (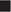), BY4741(*yrr1*Δ, pJFE1-*YRR1*) (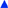), and BY4741(*yrr1*Δ, pJFE1) (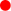) in liquid Sc-ura medium containing 6 mM vanillin.** Data are shown as the average value ± standard deviation of the biological triplicates.

To confirm that deletion of *YRR1* was the decisive factor in vanillin detoxification, compensation of *YRR1* was performed. *YRR1* was inserted into the centromere plasmid pJFE1 under the control of the *TEF1* promoter and the *PGK1* terminator, producing the plasmid pJFE1-*YRR1*. Then, pJFE1-*YRR1* was transformed into the BY4741(*yrr1*Δ), resulting in the *YRR1-*compensated strain BY4741(*yrr1*Δ, pJFE1-*YRR1*). Strains BY4741(*yrr1*Δ, pJFE1) and BY4741(pJFE1) were also constructed and used as controls. The growth test result showed that the lag phase was much longer in BY4741(*yrr1*Δ, pJFE1-*YRR1*) and BY4741(pJFE1), which expressed *YRR1*, than in BY4741 (*yrr1*Δ, pJFE1). The maximum growth rates of BY4741(pJFE1), BY4741(*yrr1*Δ, pJFE1-*YRR1*), and BY4741(*yrr1*Δ, pJFE1) were 0.0867 ± 0.0014, 0.0887 ± 0.0031, and 0.1214 ± 0.0012, respectively. The compensation of *YRR1* prolonged the lag phase and the strain growth rate decreased by 27% in liquid Sc-ura with 6 mM vanillin (**Figure 3**).

**FIGURE 4:**
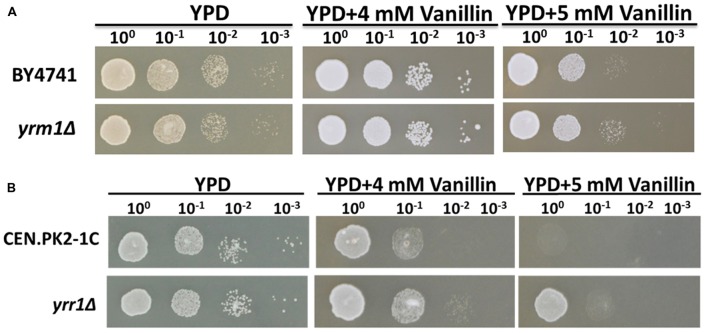
**Vanillin resistance profiles of** **(A)** BY4741 and BY4741(*yrm1*Δ) and **(B)** CEN.PK2-1C and CEN.PK2-1C(*yrr1*Δ). Four microliters of each 10-fold dilution with an initial cell OD_600_ = 1 was spotted on YPD (for 1 days) or YPD containing vanillin (for 2–3 days).

To investigate whether the deletion of *YRR1* produced a general effect on vanillin tolerance, the strain CEN.PK2-1C (*yrr1*Δ), which has a genetic background different from that of BY4741, was constructed. This strain also exhibited obviously higher vanillin tolerance compared to the parent strain CEN.PK2-1C (**Figure 4A**). This result suggested that *YRR1* deletion may be an effective method for enhancing the vanillin tolerance of *S. cerevisiae*.

Yrr1p is a transcription factor containing a Zn_2_-Cys_6_ zinc-finger motif. *YRR1* is a paralog of *YRM1*, which also contains a Zn_2_-Cys_6_ zinc-finger motif. To investigate whether Yrm1p has a function in vanillin tolerance similar to that of Yrr1p, the effect of *YRM1* deletion was also studied. The deletion of *YRM1* did not improve vanillin resistance (**Figure 4B**), demonstrating that this protein has a different function from Yrr1p in this context.

### Transcriptome Analysis of BY4741 and BY4741(*yrr1*Δ) with and without Vanillin

To investigate the mechanism by which Yrr1p influences vanillin tolerance, the transcriptomic differences between BY4741 and BY4741(*yrr1*Δ) were analyzed in SD with 5 mM vanillin or without vanillin. The genes showing significant differential regulation under different conditions are listed in **Table 2**. Genes that are underlined are down-regulated; the others are up-regulated. The genes for which 2 ≥ fold change ≥ 1.5 are marked with “^∗^”. Other genes listed in the table are those for which fold change ≥ 2.

**Table 2:** The vanillin response genes and the genes significantly regulated by Yrr1p with and without vanillin stress.

Function	BY4741 vs. BY4741(*yrr1*Δ)	BY4741 in response to 5 mM vanillin	BY4741(*yrr1*Δ) in response to 5 mM vanillin	BY4741 *vs.* BY4741(*yrr1*Δ) in the presence of 5 mM vanillin in the culture medium
Ribosome biogenesis	None	*BRX1, MAK16, NOP12, NOP4, PUF6, SSF1,BFR2, NEW1,NSR1,UTP5*	*ERB1, HAS1, IMP4, MPP10, NOB1, NOG1, NOP2, NOP7, SDA1*	*BMS1, DIP2, MPP10, NAN1, NOG1, NOG2, NOP4, NOP58, NUG1, PWP2, RIO1, SNU13, UTP13, UTP14, UTP18, UTP21, UTP4, UTP5, UTP9, NMD3, MPP10*
rRNA processing	None	*BFR2, DBP2, MAK16, NOP12, NOP4, NSR1, RRP17, SSF1, UTP5*	*DBP10, DBP2, DBP3, DRS1, ERB1, ESF1, HAS1, IMP4, IPI3, KRI1, LRP1, MPP10, MRD1, MTR4, NAN1, NOB1, NOC4, NOG1, NOP2, NOP7, NSR1, NUG1, PRP43, PWP1, RIO2, RLP7, RPF1, RRP8, SAS10, SPB1, SSF1, TSR1, TSR4*	*BFR2, BMS1, DBP10, DBP2, DBP3, DBP9, DIP2, DRS1, ENP1, ERB1, HAS1, HCA4, IPI3, MAK16, MPP10, MRD1, MTR3, MTR4, NAN1, NOC3, NOC4, NOG1, NOP12, NOP14, NOP2, NOP4, NOP58, NOP7, NSA2, NSR1, NUG1, PRP43, PUS7, PWP1, PWP2, RIO1, RIX1, RPF1, RPF2, RRP12, RRP17, RRP8, SAS10, SNU13, SOF1, SPB1, SSF1, TSR1, UTP13, UTP14, UTP18, UTP21, UTP30, UTP4, UTP5, UTP9*
Dehydrogenase	None	*ADH7, OYE2, ADH1, YPL088W*	*ADH7, OYE2, ADH2, YPL088W ADH1*	*ADH7*
ATP-binding cassette (ABC) transporter	None	*PDR5, SNQ2, YOR1^∗^*	*PDR5, SNQ2^∗^, YOR1*	*PDR5^∗^*
Other	*FMP45, YBR230W-A, YCL048W-A, SCS3, CAR2, UTH1, YIL002W-A,PMP3*	*HXT1, HXT2, HXT3, HXT4, HSP30, CMK2*	*HXT1, HXT2, HXT3, HXT4, HSP30, CMK2*	*TIF4631, NIP1*


*^∗^The genes for which 2 ≥ fold change ≥ 1.5.*

**Table 3:** Maximum specific growth rate and specific consumption rate for vanillin in *yrr1*Δ and BY4741.

Strains	SD medium	SD with 5 mM vanillin		SD with 6 mM vanillin	
			
	μ_max_ (h^-1^)	μ_max_ (h^-1^)	Specific consumption rate for vanillin (g g^-1^ h^-1^)	μ_max_ (h^-1^)	Specific consumption rate for vanillin (g g^-1^ h^-1^)
BY4741	0.355 ± 0.003	0.144 ± 0.003	0.044 ± 0.002	0.064 ± 0.001	0.055 ± 0.021
*yrr1*Δ	0.351 ± 0.010	0.178 ± 0.001	0.061 ± 0.001	0.155 ± 0.004	0.083 ± 0.007


**FIGURE 5:**
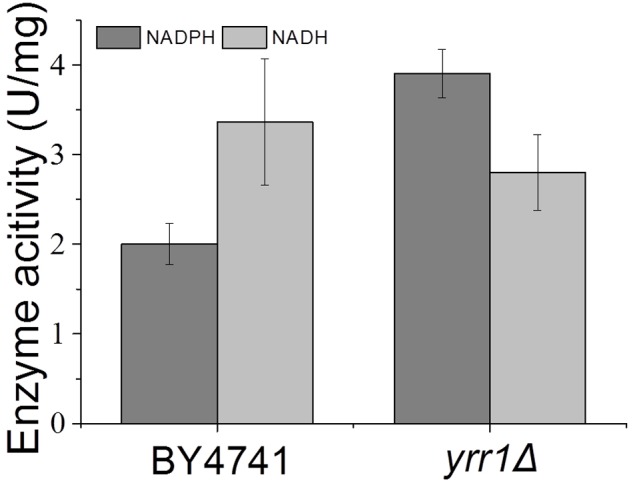
**Crude enzyme activities using vanillin as the substrate.** Error bars indicate the standard deviation of three independent replicates.

When the cells were cultured in SD medium with no vanillin stress, the transcriptome differences between BY4741 and BY4741(*yrr1*Δ) were very small. Only eight genes showed obviously different expression (**Figure 5**), and these genes do not share any functions. Next, we analyzed how the strains responded to vanillin stress. BY4741 and BY4741(*yrr1*Δ) gave similar response, for each strain, over 200 genes showed differential expression when vanillin was present compared to vanillin free condition. When vanillin was present, dehydrogenases and ATP-binding cassette (ABC) transporters were up-regulated in both strains. This may be a general mechanism for resistance to vanillin stress in *S. cerevisiae*. Interestingly, genes involved in ribosome biogenesis and rRNA processing were significantly down-regulated in BY4741, while these genes and other related genes were significantly up-regulated in BY4741(*yrr1*Δ). This result suggested that the vanillin may decrease cell growth by repressing ribosome biogenesis and rRNA processing, thereby decreasing protein synthesis, and deleting *YRR1* may restore these processes. The marked up-regulation of ribosome biogenesis and rRNA processing in BY4741(*yrr1*Δ) compared with BY4741 in the presence of 5 mM vanillin in the culture medium also supports this model. Based on these findings, more experiments were performed to further investigate how Yrr1p affects the vanillin tolerance of *S. cerevisiae*.

### *ADH7* Expression Was Induced by Vanillin, Especially in the Absence of *YRR1*

**FIGURE 6:**
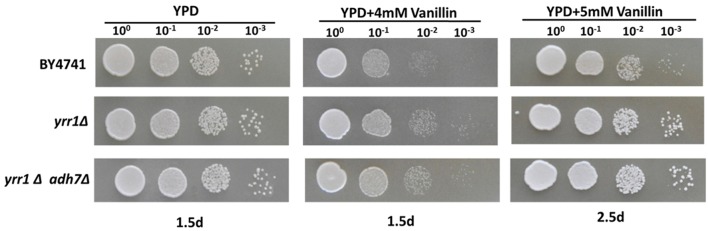
**Vanillin resistance profiles of BY4741, *yrr1Δ*, and *yrr1Δadh7*Δ.** Each test was repeated three times.

Adh7p, a NADPH-dependent medium-chain alcohol dehydrogenase, has been reported to have reductase activity and to convert vanillin to vanillyl alcohol (Nguyen et al., 2015). In both BY4741 and BY4741(*yrr1*Δ), the expression of *ADH7* was up-regulated in the presence of vanillin, which was consistent with the report that *ADH7* was inducible under severe vanillin stress (Nguyen et al., 2015). However, in BY4741(*yrr1*Δ), the increase in *ADH7* was eight times that in BY4741. The deletion of *YRR1* significantly increased the specific consumption rate of vanillin by 39 and 51% in SD with 5 and 6 mM vanillin, respectively (**Figure 2**). In BY4741(*yrr1*Δ), 6 mM vanillin was completely reduced within 26 h, while at that time point, only ∼10% of the vanillin was reduced by BY4741. Furthermore, in the enzyme activity assay, the cell-free extract of the BY4741(*yrr1*Δ) exhibited 95% higher NADPH-dependent reductase activity toward vanillin than did BY4741, while the cell-free extract of the BY4741(*yrr1*Δ) exhibited no NADH-dependent reductase activity compared to the control (**Figure 5**). Together, these results suggested that the up-regulation of *ADH7* might be one reason why deleting *YRR1* enhanced the strain tolerance to vanillin. To verify whether this is the only reason, we constructed the BY4741(*adh7Δyrr1*Δ). Interestingly, the *adh7Δyrr1*Δ strain still grew better than BY4741 in the presence of vanillin (**Figure 6**). Therefore, there are other mechanisms related to vanillin tolerance in the BY4741(*yrr1*Δ).

### Deleting *YRR1* Increased the Vanillin Tolerance of the Strain via the Increased Expression of Multidrug Transporters

**FIGURE 7:**
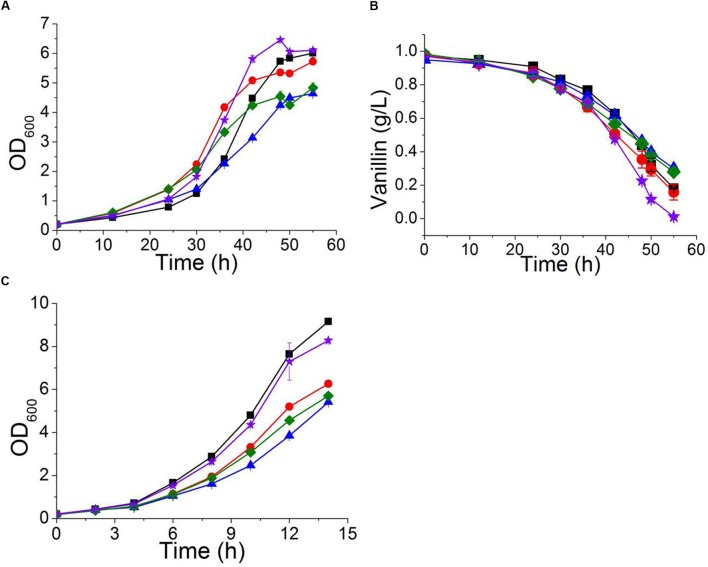
**The characteristics of strains overexpressing *PDR5* (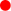); *PDR10* (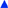); *SNQ2* (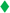); and *YOR1* (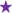), as well as the control BY4741(pJFE3) (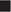) in Sc-ura with 6 mM vanillin** **(A,B)** and in Sc-ura without vanillin **(C)**. Data are shown as the mean ± standard deviation of independent triplicate experiments.

The multidrug resistance proteins Pdr5p, Yor1p, and Snq2p occur in the plasma membrane and belong to the ABC transporter superfamily, members of which export a diverse range of intracellular compounds using the energy supplied by ATP hydrolysis (Ananthaswamy et al., 2010; Guo et al., 2012). The transcriptional levels of *PDR5, YOR1*, and *SNQ2* in both BY4741 and BY4741(*yrr1*Δ) were significantly up-regulated under vanillin stress, and the degree of up-regulation of *PDR5* in BY4741(*yrr1*Δ) was notably greater than that in BY4741. Moreover, in our previous transcriptome analysis comparing EMV-8 with NAN-27, several multidrug transporters were expressed at markedly higher levels in EMV-8 than in NAN-27, including Pdr10p (6.2 times) and Yor1p (3.6 times). Next, the abovementioned genes related to multidrug resistance were overexpressed in the lab strain BY4741. In Sc-ura medium, the strain overexpressing *PDR5* and *PDR10* grew much more slowly than the control BY4741(pJFE3) (**Figure 7C**). Nonetheless, when vanillin was present, the strain overexpressing *PDR5*, *YOR1*, and *SNQ2* grew markedly faster than control (**Figures 7A,B**) and had a shorter lag phase which was defined as the time before OD_600_ reached 1.0 (**Table 4**).

**Table 4:** Maximum specific growth rate and specific consumption rate for vanillin of recombinant strains.

Strains	SD medium	SD with 6 mM vanillin		
		
	μ_max_ (h^-1^)	μ_max_ (h^-1^)	Specific consumption rate for vanillin (g g^-1^ h^-1^)	Lag phase^∗^ (h)
BY4741(pJFE3)	0.290 ± 0.005	0.0980 ± 0.0009	0.035 ± 0.001	∼30
BY4741(*PDR5*)	0.2190 ± 0.008	0.0808 ± 0.0008	0.030 ± 0.002	<24
BY4741(*SNQ2*)	0.208 ± 0.000	0.0704 ± 0.0001	0.029 ± 0.002	∼24
BY4741(*YOR1*)	0.290 ± 0.009	0.0828 ± 0.0011	0.035 ± 0.002	∼24
BY4741(*PDR10*)	0.208 ± 0.007	0.0773 ± 0.0011	0.037 ± 0.001	∼24


*^∗^The time when OD_*600*_ reached 1.0.*

### Deleting *YRR1* Increased the Vanillin Tolerance of the Strain via the Enhancement of Ribosome Biogenesis and rRNA Processing

**FIGURE 8:**
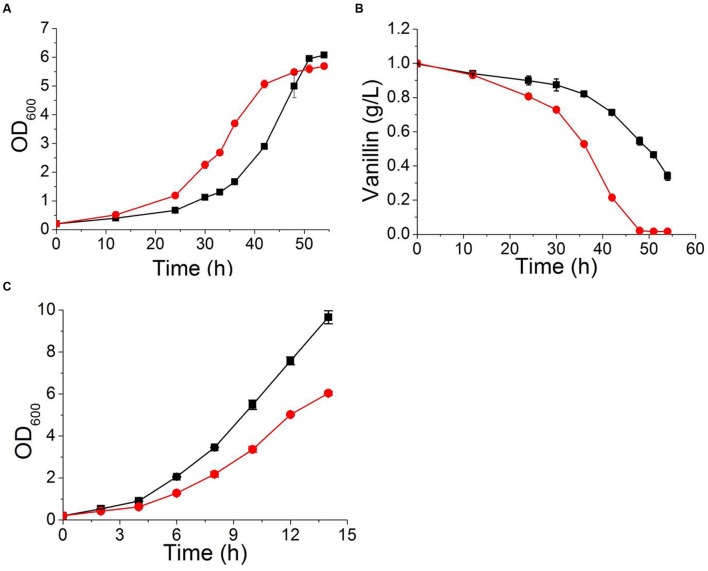
**The characteristics of a strain overexpressing *DBP2* (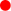) and the control strain BY4741(pJFE1) (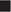) in Sc-ura containing 6 mM vanillin** **(A,B)** and in Sc-ura without vanillin **(C)**. Data are shown as the mean ± standard deviation of the biological triplicates.

Ribosomes are responsible for protein synthesis in the cell (Pelava et al., 2016). Reduced or abnormal ribosome production occurs in response to cellular stress and leads to decreased growth and metabolic capacity of the strain (Nomura, 1999). Our transcriptional analysis result indicated that ribosome biogenesis and rRNA processing were repressed by vanillin in wildtype *S. cerevisiae*. Deleting *YRR1* increased the expression of genes related to ribosome biogenesis and rRNA processing when vanillin was present. Nine RNA helicases, including *DBP2*, *DBP3*, *DBP9, DBP10, PRP43, MTR4, HAS1*, *DHH1*, and *DRS1*. It was reported that the proteins encoding by these genes are participated in ribosome biogenesis, rRNA processing, and translation initiation (Rodriguez-Galan et al., 2013), were identified from dozens of markedly up-regulated genes in the comparison between BY4741 and BY4741(*yrr1*Δ). To further confirm whether enhancing ribosome biogenesis and rRNA processing can increase the vanillin tolerance of a strain, the gene *DBP2*, which encodes an ATP-dependent RNA helicase that functions in mRNP assembly (Ma et al., 2013) and is the gene most significantly down-regulated in BY4741 under vanillin stress but up-regulated in *yrr1*Δ, was overexpressed in BY4741. Although the maximum specific growth rate of BY4741(*DBP2*) was decreased by 15% compared to the control under vanillin-free conditions, in the presence of vanillin, the growth and metabolic characteristics of BY4741(*DBP2*) were better than those of the control. In SD medium containing 6 mM vanillin, the maximum specific growth rate of strain overexpressing *DBP2* was 7% higher than that of the control. The OD_600_ of the *DBP2*-overexpressing strain reached 1.0 and 2.0 after only 24 and 30 h, respectively, while reach the same biomass in the control required 30 and 40 h, respectively. The specific vanillin consumption rate of the strain overexpressing *DBP2* was 0.062 ± 0.002 g g^-1^ h^-1^, which was 59% higher than that of the control (0.039 ± 0.001 g g^-1^ h^-1^, **Figure 8**).

## Discussion

Yrr1p, a Zn_2_-Cys_6_ zinc-finger transcription factor, has been reported to be an activator of genes related to multidrug resistance (Zhang et al., 2001; Le Crom et al., 2002; Teixeira et al., 2008; Kodo et al., 2013). It has been reported that Yrr1p contributes to tolerance of toxicants such as 4-nitroquinoline-*N*-oxide (4-NQO) (Cui et al., 1998). In the present work, we found that the absence of Yrr1p provided yeast strains with vanillin resistance characteristics.

The transcription result showed that only eight genes (**Table 5**) were significantly differentially regulated after *YRR1* deletion when the cells were cultured under vanillin-free conditions. Furthermore, changing the expression levels of these genes had no effect on the vanillin tolerance of strain (data not shown). However, the situation was quite different when strains were cultured under vanillin stress. When vanillin was present, the absence of Yrr1p led to a marked effect on the transcriptome, with at least three related functions: vanillin detoxification, excretion of vanillin or other toxic molecules generated because of vanillin stress, and the promotion of the biological processes of rRNA processing and ribosome biogenesis. In these up-regulated genes, the dehydrogenase gene *ADH7* was the most up-regulated in comparison of BY4741 and BY4741(*yrr1*Δ) when vanillin existed. Adh7p and its homologous gene Adh6p were considered to reduce vanillin to vanillyl alcohol (Liu, 2011; Wang et al., 2016). However, the deletion of *ADH7* in BY4741(*yrr1*Δ) didn’t affect its tolerance to vanillin. Hence, *ADH7* was not the main reason why deleting *YRR1* enhanced the strain tolerance to vanillin. Genes for dehydrogenases and ABC transporters were up-regulated under vanillin stress. When Yrr1p was absent, their expression levels were even higher. Genes involved in ribosome biogenesis and rRNA processing, which are important for protein synthesis, were down-regulated under vanillin stress but up-regulated when Yrr1p was absent. These phenomena suggested that vanillin might be an effector molecule in this context. Vanillin may induce the expression of some genes related to rRNA processing and ribosome biogenesis. However, these genes are directly or indirectly repressed by Yrr1p, only when Yrr1p is absent, their expression are activated by vanillin. The regulatory mechanism of Yrr1p under vanillin stress remain to be discovered.

**Table 5:** The genes significantly regulated after deletion of *YRR1* without vanillin stress.

Gene name	Functions	Expression regulation
*YBR230W-A*	Putative protein of unknown function; *YBR230W-A* has a paralog, *COQ8.*	up
*CAR2*	*L*-ornithine transaminase (OTAse).	up
*FMP45*	Integral membrane protein localized to mitochondria; required for sporulation and maintaining sphingolipid content.	up
*YCL048W-A*	Putative protein of unknown function.	up
*SCS3*	Protein required for inositol prototrophy; required for normal ER membrane biosynthesis.	up
*UTH1*	Mitochondrial inner membrane protein.	up
*YDR276C*	Small plasma membrane protein.	down
*YIL002W-A*	Putative protein of unknown function.	down


ATP-binding cassette transporters have been considered to play a key role in increasing strains’ tolerance to chemicals by exporting inhibitors (Liu, 2011). In the present study, overexpression of *PDR5*, *YOR1*, and *SNQ2* was demonstrated to improve vanillin tolerance. However, overexpressing these large proteins also burdened the cells. The strain overexpressing these genes grow more slowly than wildtype under vanillin-free conditions or after vanillin was depleted. Therefore, their expression should be controlled at a suitable level.

When cells are stimulated by harmful conditions, such as starvation, heat shock, oxidative stress, or acidic stress, translation is depressed (Nomura, 1999; Shenton et al., 2006; Hoyle et al., 2007; Iwaki and Izawa, 2012; Iwaki et al., 2013a; Dever et al., 2016). It has been reported that under the vanillin stress, messenger ribonucleoprotein (mRNP) granule formation (P-bodies and stress granules) may form because the initiation of translation was inhibited and the polysomes were disassembled (Iwaki et al., 2013b). Our transcription results also confirmed that ribosome biogenesis and rRNA processing, which are considered the source of all protein synthesis (Pelava et al., 2016), were significantly inhibited by vanillin. Furthermore, up-regulating ribosome biogenesis and rRNA processing, whether by deleting *YRR1* or overexpressing *DBP2*, improves cell growth under vanillin stress. Only overexpressing single or several genes did not elevate the protein synthesis efficiently, whereas deleting *YRR1* holistically enhanced the ribosome biogenesis and rRNA processing.

## Conclusion

We compared the genome sequence of a vanillin-tolerant strain and its parent. Then, we found that the loss of Yrr1p is an efficient strategy for enhancing the vanillin tolerance of *S. cerevisiae*, likely by improving the strain’s capacity for vanillin detoxification, vanillin excretion, rRNA processing and ribosome biogenesis. Furthermore, the up-regulation of rRNA processing and ribosome biogenesis occurred only in the presence of vanillin, suggesting that vanillin might be an effector molecule in this regulatory process. To our knowledge, this is the first report of a relationship between *YRR1* and vanillin tolerance and the first report of enhancing *S. cerevisiae* vanillin tolerance via up-regulating rRNA processing and ribosome biogenesis. These new findings will provide novel targets for constructing strains tolerant of lignocellulosic materials and valuable insights into the novel functions of *YRR1* in *S. cerevisiae*.

## Author Contributions

XW, YS, and XB conceived and designed the study; XW and ZL participated in the design of experiments and data collection; XW performed genome sequencing and RNA-seq data analysis; XW and YS analyzed data and drafted the manuscript. YS, XB, and JH supervised and coordinated the overall study. All authors read and approved the final manuscript.

## Conflict of Interest Statement

The authors declare that the research was conducted in the absence of any commercial or financial relationships that could be construed as a potential conflict of interest.

## Footnotes

1 http://sgd-beta.stanford.edu/

## Abbreviations

NADH: Reduced form of nicotinamide adenine dinucleotide
NADPH: Reduced form of nicotinamide adenine dinucleotide phosphate
ROS: reactive oxygen species
